# The developmental effects of HIV and alcohol: a comparison of gestational outcomes among babies from South African communities with high prevalence of HIV and alcohol use

**DOI:** 10.1186/s12981-017-0153-z

**Published:** 2017-05-08

**Authors:** Kirsten A. M. Donald, Anne Fernandez, Kasey Claborn, Caroline Kuo, Nastassja Koen, Heather Zar, Dan J. Stein

**Affiliations:** 1 Department of Paediatrics and Child Health, Institute of Child Health Building, Red Cross War Memorial Children’s Hospital, 5th Floor, Klipfontein Road, Rondebosch, Cape Town, 7700 South Africa; 2 Center for Alcohol and Addiction Studies, Brown University School of Public Health, Providence, RI USA; 3 Department of Medicine, Rhode Island Hospital, Providence, USA; 4 Department of Psychiatry and Human Behavior, Warren Alpert Medical School of Brown University, Providence, USA; 5 Department of Behavioral and Social Sciences, Brown University School of Public Health, Providence, RI USA; 6 Department of Psychiatry and Mental Health, University of Cape Town, Cape Town, South Africa; 7 South African Medical Research Council Unit on Child & Adolescent Health, Cape Town, South Africa; 8 South African Medical Research Council Unit on Risk and Resilience in Mental Disorders, Cape Town, South Africa

**Keywords:** Gestational outcomes, Alcohol, HIV, South Africa, Pregnancy

## Abstract

**Background:**

There is growing evidence of the negative impact of alcohol on morbidity and mortality of individuals living with HIV but limited evidence of in utero effects of HIV and alcohol on exposure on infants.

**Methods:**

We conducted a population-based birth cohort study (N = 667 mother-infant dyads) in South Africa to investigate whether maternal alcohol use and HIV affected gestational outcomes. Descriptive data analysis was conducted for all variables using frequency distributions, measures of central tendency, and estimates of variance. Hierarchical multiple regression was conducted to determine whether maternal alcohol use, maternal HIV status and other risk factors (socioeconomic status, smoking, depression) predicted infant outcomes.

**Results:**

Our results showed severity of recent alcohol use and lifetime alcohol use predicted low birth weight. Similarly lifetime alcohol use predicted shorter infant length, smaller head length, smaller head circumference, and early gestational age. However, HIV status was not a significant predictor of gestational outcomes.

**Conclusions:**

The unexpected finding that maternal HIV status did not predict any of the gestational outcomes may be due to high rates of ART usage among HIV-infected mothers. The potentially negative effects of HIV on gestational outcomes may have been attenuated by improved maternal health due to high coverage of antiretroviral treatment in South Africa. Interventions are needed to reduce alcohol consumption among pregnant mothers and to support healthy growth and psychosocial development of infants.

## Background

Alcohol use, including hazardous and harmful levels, remains a serious health problem among individuals living with HIV globally and in South Africa specifically. In South Africa, the country with the largest number of people living with HIV (PLWH) globally [1], alcohol consumption is among the highest in the world [2]. A national survey documented that 9% of the population engage in risky or hazardous drinking [3–5]. Even more concerning is a high proportion of individuals living with HIV struggle with alcohol abuse and dependence. While national estimates are unavailable, local studies in communities with high prevalence of HIV show rates of recent alcohol use as high as 51% as well as concerning levels of dependence or abuse among PLWH (7%) [6, 7].

A growing body of literature highlights the synergistic and detrimental effects of alcohol on morbidity and mortality of individuals living with HIV [8]. Alcohol use accelerates disease progression via biological pathways. This includes both increased viral replication and diminished immune function. For example, alcohol use is associated with depletion of CD4 cells [9, 10] and has been shown to negatively affect performance of T and B lymphocytes and other important immune responses [11–15]. Alcohol use also accelerates disease progression via behavioral pathways for PLWH. A recent meta-analysis and a systematic review showed that alcohol use was linked to poorer adherence to antiretroviral therapy and lower utilization of health services [16, 17].

The negative health-related consequences of alcohol use among PLWH may be of even greater concern among pregnant women living with HIV. Several meta-analyses indicate that heavy alcohol use during pregnancy can increase risk for low birth weight, preterm birth, small gestational age [18] and long term growth impact including gross motor deficits [19]. Similarly, maternal HIV infection has been associated with poor fetal outcomes including low birth weight [20] and pre-term delivery [21, 22]. Maternal HIV infection, particularly mother-to-child transmission of HIV, can affect fetal development of the infant brain. For example, there is growing evidence that babies infected with HIV are at risk of significantly poorer performance of infant motor and cognitive development even if they do not meet criteria for HIV encephalopathy [23]. There may be synergistic effects of alcohol and HIV on health, particularly during the gestational period. As such, additional research is needed to determine whether HIV might interact with alcohol to negatively affect infant outcomes among babies born to mothers affected by HIV and using alcohol.

South Africa is an ideal setting to investigate whether living with HIV and using alcohol during pregnancy might have synergistic affects upon fetal outcomes. Rates of alcohol use during pregnancy have been well-documented in South Africa. South Africa has the highest global rates of fetal alcohol syndrome (FAS) [24]. However, only a handful of studies document rates of alcohol use among HIV-infected pregnant women in South Africa. One cross-sectional survey of pregnant women attending clinics documented that 18 percent engaged in drinking during pregnancy and 67% reported problem drinking (more than 3 drinks in one sitting) [25]. Another study analyzing a subset of data from a longitudinal cohort of female drinkers in Cape Town documented that 73% continued drinking after recognition of pregnancy [26]. However, no study to date has compared alcohol use patterns among HIV-positive and HIV-negative pregnant women or examined whether alcohol use among pregnant mothers living with HIV has any effect on gestational outcomes.

We conducted a population-based birth cohort study in the Western Cape Province, a region supported heavily by an agricultural economy centered on wine production. Our choice of this site was purposeful given the rates of alcohol abuse and dependence, and HIV prevalence in this region. Rates of alcohol use may be particularly problematic due to the historical legacy of the ‘dop’ system where farm workers were paid in crude wine [27]. Rates of FAS or Partial Fetal Alcohol Syndrome (PFAS) are alarmingly high, at 135–207 cases per 1000 people in certain high risk communities in the Western Cape [28]. Our analyses focused on determining whether there were differences in alcohol intake between HIV-positive and HIV-negative pregnant women and whether HIV or alcohol had any synergistic effects of gestational outcomes of birth weight percentile, length, head circumference, and gestational age.

## Methods

Data was collected from an investigation of child health, called the Drakenstein Child Health Study (DCHS). The DCHS consisted of a multidisciplinary birth cohort study following mother–child dyads from the prenatal period through to five years of age [29, 30]. The aim of DCHS is to investigate the epidemiology, etiology, and risk factors of childhood respiratory disease and the determinants of child health in a low socioeconomic area of South Africa [30]. The study investigates the role and interaction of possible risk factors in 7 areas (environmental, infectious, nutritional, genetic, psychosocial, maternal and immunological risk factors) that may impact child health.

### Design

Pregnant women were recruited from two primary care clinics in the Drakenstein sub-district near Cape Town, South Africa between March 2012 and September 2014. Data collection occurred at these two clinics (maternal data) and at a central hospital (newborn outcomes). Participants were enrolled in the study at 20–28 weeks gestation upon presenting for antenatal booking. The mothers were followed throughout pregnancy and mother–child dyads are followed until five years after birth. Data collection for the DCHS remains ongoing. Throughout the study, participants receive regular medical care as per the South Africa national program and expanded program for immunization (EPI) schedule.

### Setting

The DCHS is located in the Drakenstein area, a peri-urban area, 60 km outside Cape Town, South Africa with a population of approximately 200,000. The community is stable, with low levels of immigration or emigration. The local economy is based around commercial agriculture and light industry. More than 90% of the population access health care in the public sector including antenatal and child health services [31]. This area has a well-established, free primary health care system, with high coverage for childhood immunizations. The public health system is comprised of 23 primary health care clinics and one centralized hospital where all births and all hospital-based pediatric care, including admissions occur [30].

### Participants

To be eligible for the study, participants were: (a) over age 18, (b) pregnant, (c) 20–28 weeks gestation, and (d) receiving antenatal care at a participating study site. The two primary clinics were TC Newman clinic (serving a predominantly “Coloured” or mixed ancestry community) and Mbekweni clinic (serving a predominantly black African community). Exclusion criteria were minimal in order to maximize generalizability, and focused primarily on those individuals who did not live in the region (and could not readily complete follow-up assessments) or those who were intending to move out of the district within the following 2 years. The cohort comprised mothers presenting in an unfiltered manner to the antenatal services and were not selected for any clinical risk factors or exposures. The study was approved by the Faculty of Health Sciences, Human Research Ethics Committee, University of Cape Town (401/2009), and by the Western Cape Provincial Health Research committee. Written informed parental consent was obtained from all participants at the time of enrolment.

### Measures

#### Demographics

Demographic data was collected regarding participant age, education, employment, and socioeconomic status (SES). For our purposes, a composite SES score was developed in order to categorize participants into quartiles based on their relative SES. This composite score is calculated based on current employment status and standardized scores of educational attainment, household income, and a composite asset index based on access to household resources, amenities, and market access. Participants were categorized as being of low, low-moderate, moderate-high, or high SES based on their relative composite SES score.

#### HIV status

Maternal HIV status was determined using an HIV ELISA test, which all mothers in South Africa receive during pregnancy due to the high prevalence of HIV. Those who tested positive for HIV self-reported whether she was prescribed antiretroviral therapy. Mothers who were newly diagnosed received a second confirmatory HIV ELISA test and were referred for counseling and evaluation for treatment initiation as per standard management guidelines.

#### Newborn birth outcomes

All babies were born at a central hospital that serves the communities of both clinics. Trained clinical staff recorded newborn birth weight (kilograms) at the time of delivery, using a digital scale with a precision level of 10 g. Gestational age (weeks), head circumference and length in centimeters were also recorded at birth. We defined low birth weight and very low birth weight according to World Health Organization’s (WHO’s) parameters for weight for age, with low birth weight defined as less than 2.5 kilograms and very low birth weight as less than 1.5 kg [32]. Percentile for infant birth weight was then calculated using the formula proposed by Mikolajcyzk et al. [33]. This formula enabled us to calculate the birth weight percentile based on gestational week of each infant. We used full-term, low-risk infants (N = 100) as the reference group for anchoring our percentile estimates. This method for estimating fetal weight percentiles has a better ability to predict adverse perinatal outcomes than other unadjusted and fully individualized formulas [33].

#### Alcohol use

Risk of substance use was assessed using the alcohol, smoking and substance involvement screening test (ASSIST) at the second antenatal visit (28–32 weeks gestation). This tool was developed by the WHO to detect psychoactive substance use and related disorders in primary care settings. It has shown good reliability, feasibility and validity in international, multisite studies [34, 35], and is useful for identifying risk of substance dependence in poly-substance abusers with varying degrees of psychopathology. Seven items are included to assess alcohol and other drug use across 10 categories (i.e., tobacco products; alcoholic beverages; cannabis; cocaine; amphetamine-type stimulants; inhalants, sedatives or sleeping pills; hallucinogens; opioids; and a general category entitled “other”, in which the participant is required to specify the substance used). ASSIST scores from 0 to 10 for alcohol and 0–3 for illicit drugs indicating low-risk, scores from 11 to 26 for alcohol and 4–26 for illicit drugs indicate moderate-risk and scores above 26 indicate high risk of severe problems, with the likelihood of substance dependence. The higher the score, the greater substance-related risk. The ASSIST has good reliability scores and has been validated in numerous populations [36], as well as primary care settings [37]. For our purposes, only data for alcohol were extracted from the ASSIST, as reports of other substance use were negligible with the exception of tobacco where a biochemical measure of exposure was used as detailed below. Lifetime alcohol use was a dichotomous variable coded as 0, 1. Past 3 month alcohol use severity was calculated as a continuous variable using standard scoring techniques to calculate a sum score based on past 3-month alcohol use frequency, craving, consequences, and other symptoms of alcohol use disorders as detailed above [38].

#### Tobacco use

Biochemical verification of smoking status was determined through cotinine urine analysis collected on the same day as the baseline interview. Cotinine is a nicotine metabolite that is measurable among people who smoke, use smokeless tobacco, use nicotine products, or are exposed to environmental tobacco smoke. An antenatal urine cotinine cut-off value of 500 was used to distinguish active smokers from non-smokers [39].

#### Depression

The Beck Depression Inventory-II (BDI-II) [40] was used to assess depressive symptomatology. This is a widely used self-report measure of the severity of depressive symptoms with strong internal consistency and concurrent validity in clinical and non-clinical samples [41, 42]. Each item is assessed on a severity scale ranging from 0, “absence of symptoms”, to 3 “severe, often with functional impairment”. A total score was obtained by summing individual item responses, with higher scores indicative of more severe depressive symptoms.

### Data analysis

Descriptive data analysis was conducted for all variables using frequency distributions, measures of central tendency, and estimates of variance. Hierarchical multiple regression was conducted to determine whether maternal HIV status, alcohol use, and other risk factors (socioeconomic status or SES, smoking, depression) predicted infant outcomes. Infant outcomes included birth weight percentile, length, head circumference, and gestational age. All outcomes were entered as continuous variables in regression analyses. The analyses were conducted in a step-wise fashion to examine the unique contributions for each block of predictor variables. The four steps/blocks of variables in the regression analyses included (in order): (1) HIV status, (2) SES score, BDI-II score, and smoking status, (3) alcohol use, and (4) interaction variables (HIV by alcohol use and HIV by smoking status). We examined separate models using the predictors past 3-month alcohol use severity and lifetime alcohol. All regression models were examined for violations of normality, linearity, homoscedasticity, redundancy, and multicollinearity. Data performed excellently on all criteria except for length of gestation. Length of gestation was non-normal (skew = −2.04, kurtosis = 7.38) and the log of this variable was used in the analysis. Statistical significance was set at the 0.05 level.

## Results

### Descriptive analyses

The sample included 667 mother-infant dyads of whom 19.6% (n = 131) of the maternal sample was HIV-positive (Table 1). All mothers with HIV were on ART; no infants were HIV-infected. A diverse range of socioeconomic backgrounds was represented, and 26.7% (n = 178) of mothers were categorized as low SES. In terms of depression as measured by the BDI-II, 25% (n = 167) of the mothers reported symptoms consistent with mild depression, 22.5% (n = 150) reported moderate depression, and 7.8% (n = 52) reported high/severe depression. With regards to alcohol use as measured by the ASSIST, 42.2% (n = 280) of mothers reported a lifetime history of alcohol use, and 21.54%, (n = 148) reported alcohol use in the past 3-months. Heavy alcohol use in the past 3-months was reported by a small percentage of mothers (3.1%; n = 21), moderate alcohol use was reported by 7.7% (n = 53), and light use by 10.8% (n = 74). In this cohort, HIV-infected mothers had significantly lower lifetime history of alcohol use (14.6%) compared to HIV-negative mothers (48.9%), χ^2^ (1, *N* = 664) = 50.32, *p* < 001; lower 3-month alcohol use (light use: 12.8% vs. 10.8%, moderate use: 8.5% vs. 4.6%, heavy use: 2.9% vs. 3.8%, χ^2^ (1, *N* = 148) = 7.78, *p* < *0.020*. Approximately 1 in 3 mothers were current smokers (32.7%; n = 213).

**Table 1:** Maternal characteristics and risk factors by maternal HIV status

	HIV negative		HIV positive		Total		p value
	N = 556	%	N = 131	%	N = 667	%	
Socio-economic status							<0.000
Low	125	23.32	53	40.46	178	26.69	
Low-moderate	101	18.84	34	25.95	135	20.24	
Moderate-high	157	29.29	27	20.61	184	27.59	
High	153	28.54	17	12.98	170	25.49	
Depression							<0.095
Minimal	244	45.52	54	41.22	298	44.68	
Mild	137	25.56	30	22.90	167	25.04	
Moderate	120	22.39	30	22.90	150	22.49	
High	35	6.53	17	12.98	52	7.80	
Lifetime alcohol use							
Yes	261	48.88	19	14.62	280	42.17	<0.000
Past 3-month alcohol use severity score							<0.020
None	422	75.90	117	89.31	539	78.46	
Light	71	12.77	3	2.29	74	10.77	
Moderate	47	8.45	6	4.58	53	7.71	
Heavy	16	2.88	5	3.82	21	3.06	
Smoking (Cotinine)							
Yes	183	35.06	30	23.08	213	32.67	<0.009

Infant outcomes are presented in Table 2. The mean birth weight was 3.02 kilograms (SD = 0.58), with 3.3% (n = 22) of infants categorized as low birth weight (<10th percentile) and 4.8% (n = 32) of infants categorized as very low birth weight (<3rd percentile). The mean gestational age was 38.35 weeks (SD = 2.58). Pre-term infants (<37 weeks gestation) accounted for 15.6% (n = 104) of the infant sample. The mean head circumference was 33.55 cm (SD = 1.87). The mean length was 49.86 cm (SD = 3.8).

**Table 2:** Infant characteristics by maternal HIV status

	HIV negative		HIV positive		Total		p value
	N (556)	%	N (131)	%	N (667)	%	
Birth weight percentile (%)							<0.748
<3	28	5.23	4	3.05	32	4.80	
3 to <10	20	3.74	2	1.53	22	3.30	
≥10	487	91.03	125	95.42	612	91.89	
Gestational age (full/pre-term)							
<37 weeks	80	14.93	24	18.32	104	15.59	<0.337

	HIV negative		HIV positive		Total		p value
	Mean	SD	Mean	SD	Mean	SD	
Infant birth weight (kg)	3.01	0.58	3.03	0.58	3.02	0.58	<0.729
Infant head circumference (cm)	33.50	1.80	33.76	2.08	33.55	1.87	<0.944
Infant length (cm)	49.87	3.63	49.84	4.48	49.86	3.8	<0.153
Length of gestation (weeks)	38.39	2.45	38.21	3.07	38.35	2.58	<0.489

*cm* centimeters, *kg* kilograms

### Hierarchical multiple regression analyses

#### Birth weight percentile

Model results for the hierarchical multiple regression analysis for birth weight percentile is presented in Table 3. Step 1 of the model, which included HIV status was not significant, and accounted for a very small proportion of variance in birth weight percentile, F (1, 648) = 2.92, p = 0.09, *R* ^*2*^ = 0.004. Step 2, which added smoking, SES, and depression, was significant, F (4, 648) = 10.88, p < 0.001, *R* ^*2*^ = 0.06, and increased the proportion of variance accounted for by 6% (*ΔR* ^*2*^ = 0.06). Step 3, which added alcohol use severity was significant, F (5, 650) = 10.17, p < 0.001, *R* ^*2*^ = *0*.07. The change in variance accounted for was 1% (*ΔR* ^*2*^ = 0.01). Step 4, which added the interaction terms, HIV by alcohol and HIV by smoking, did not improve the model. Therefore Step 3 was chosen as the final model. The standardized beta weights for the final model are presented in Table 4. Alcohol use severity (β = −0.10), smoking (β = −0.18), and depression (β = −0.09) were significant predictors of birth weight percentile.

**Table 3:** Results of multiple regression analyses for birth weight percentile, length, head circumference, and gestational age

		df	Mean square	F value	p value	R2	Ä R2
Birth weight percentile							
Block 1	HIV status (pos/neg)	1	3004	2.92	0.088	0.00	
Block 2	Smoking, depression, SES	4	10,597	10.88	0.000	0.06	0.06
Block 3	3-month alcohol use severity	5	9816	10.17	0.000	0.07	0.01
Block 4	Interactions	7	7058	7.29	0.000	0.07	0.00
Length							
Block 1	HIV status (pos/neg)	1	0.001	0.001	0.992	0.00	
Block 2	Smoking, depression, SES	4	115.17	8.23	0.000	0.05	0.05
Block 3	3-month alcohol use severity	5	93.36	6.67	0.000	0.05	0.00
Block 4	Interactions	7	66.83	4.76	0.000	0.05	0.00
Head circumference							
Block 1	HIV status (pos/neg)	1	7.352	2.10	0.148	0.00	0.00
Block 2	3-month alcohol use severity	4	38.649	11.73	0.000	0.07	0.07
Block 3	3-month alcohol use severity	5	31.496	9.56	0.000	0.07	0.00
Block 4	Interactions	7	22.599	6.84	0.000	0.07	0.00
Gestational age (log)							
Block 1	HIV status (pos/neg)	1	0.003	0.60	0.439	0.00	
Block 2	Smoking, depression, SES	4	0.021	3.86	0.004	0.02	0.02
Block 3	3-month alcohol use severity	5	0.021	3.85	0.002	0.03	0.01
Block 4	Interactions	7	0.015	2.80	0.007	0.03	0.00

**Table 4:** Standardized beta weights and t test results for predictors of birth weight percentile

	Beta	SE	t	p value
Birth weight percentile				
Constant		2.34	24.39	<0.000
Mother’s confirmed HIV status	0.057	3.14	1.47	0.143
SES	0.046	0.59	1.16	0.247
BDI score	−0.085	0.13	−2.18	0.030
Smoker (yes, no)	−0.184	2.68	−4.71	<0.000
3-month alcohol use severity	−0.103	0.17	−2.63	0.009

*SES* socioeconomic status, *BDI* Beck Depression Inventory

We ran a subsequent model using lifetime alcohol use as a predictor in Step 3 of the regression equation. Step 1 and Step 2 model results were identical. Step 3, which added lifetime alcohol use was also significant, F (5, 648) = 11.91, p < 0.001, *R* ^*2*^ = 0.085, and increased the proportion of variance accounted for by 2% (*ΔR* ^*2*^ = 0.02). The standardized beta weight for lifetime alcohol use was −0.161 (p < 0.001). In Step 4, the interaction terms were not significant predictors of birth weight percentile.

#### Infant length

Model results for the hierarchical multiple regression analysis for infant length are presented in Table 3. Step 1 of the model, which included HIV status only was not significant, and accounted for no variance in infant length, F(1, 647) = 0.001, p = 0.99, *R* ^*2*^ < 0.000. Step 2, which added smoking, SES, and depression, was significant F (4, 647) = 8.23, p < 0.001, *R* ^*2*^ = 0.05, and increased the proportion of variance accounted for by 5% (*ΔR* ^*2*^ = 0.05). Step 3 and Step 4 did not improve the model. Smoking (β = −0.196) was the only significant predictor of infant length.

We ran a subsequent model using lifetime alcohol use as a predictor in Step 3 of the regression equation. Step 1 and Step 2 model results were identical. Step 3, which added lifetime alcohol use was also significant, F (5, 645) = 7.14, p < 0.001, *R* ^*2*^ = 0.053, and increased the proportion of variance accounted for by 0.4% (*ΔR* ^*2*^ = 0.004). The standardized beta weight for lifetime alcohol use was −0.07 (p = 0.09). In Step 4, the interaction terms were not significant predictors of infant length.

#### Head circumference

Model results for the hierarchical multiple regression analysis for head circumference are presented in Table 3. Step 1 of the model, which included HIV status only was not significant, and accounted for very little variance in head circumference, F (1, 645) = 2.10, p = 0.148, *R* ^*2*^ = 0.003. Step 2, which added smoking, SES, and depression, was significant F (4, 645) = 11.73, p < 0.001, *R* ^*2*^ = 0.07, and increased the proportion of variance accounted for by 7% (*ΔR* ^*2*^ = 0.07). Step 3 and Step 4 did not improve the model. Smoking (β = −0.21), and depression (β = −0.09) were significant predictors of head circumference.

We ran a subsequent model using lifetime alcohol use as a predictor in Step 3 of the regression equation. Step 1 and Step 2 model results were identical. Step 3, which added lifetime alcohol use was also significant, F (5, 645) = 10.35, p < 0.001, *R* ^*2*^ = 0.068, and increased the proportion of variance accounted for by 0.5% (*ΔR* ^*2*^ = 0.005). The standardized beta weight for lifetime alcohol use was −0.08 (p = 0.06). In Step 4, the interaction terms were not significant predictors of head circumference.

#### Gestational age

Model results for the hierarchical multiple regression analysis for gestational age are presented in Table 3. Step 1 of the model, which included HIV status was not significant, and accounted for very little variance in gestational age, F (1, 651) = 0.60, p = 0.44, *R* ^*2*^ = 0.001. Step 2, which added smoking, SES, and depression, was significant F (4, 651) = 3.86, p = 0.023, *R* ^*2*^ = 0.02, and improved the model, (*ΔR* ^*2*^ = 0.02). Step 3 and Step 4 did not improve the model. Only SES was significantly related to gestational age (β = 0.14).

We ran a subsequent model using lifetime alcohol use as a predictor in Step 3 of the regression equation. Step 1 and Step 2 model results were identical. Step 3, which added lifetime alcohol use was also significant, F (5, 648) = 11.91, p < 0.001, *R* ^*2*^ = 0.085, and increased the proportion of variance accounted for by 2% (*ΔR* ^*2*^ = 0.02). The standardized beta weight for lifetime alcohol use was −0.161 (p < 0.001). In Step 4, the interaction terms were not significant predictors of birth weight percentile.

## Discussion

This is the first study that examines the relationship between maternal HIV and alcohol use on infant birth outcomes in a setting with some of the highest global prevalence of HIV and alcohol use. Our results indicate that there were concerning levels of lifetime and recent alcohol use among pregnant women. In regards to alcohol use, we found lower lifetime and recent alcohol use reported by HIV-positive mothers. Recent and lifetime alcohol use predicted lower birth weight, and lifetime alcohol use predicted shorter infant length, smaller head circumference, and younger gestational age. The finding that alcohol use severity did not predict all gestational outcomes was unexpected, and is possibly due to relatively low rates of reported past 3-month alcohol use overall, and large percentages of light alcohol users among past 3-month alcohol users. The small number of heavy alcohol users documented on this self-report tool likely limited our ability to detect alcohol use on gestational outcomes. Regardless, the overall findings point towards the detrimental effects of alcohol on infant outcomes. The negative effects of alcohol on gestational outcomes borne out in our cohort are consistent with the existing literature [18].

We also examined whether maternal HIV and alcohol had any synergistic effects of gestational outcomes. Our results for this analyses showed that HIV and alcohol did not appear to have significant synergistic effects on gestational outcomes of birth weight percentile, length, head circumference, and gestational age. These findings were surprising, but should be interpreted with caution. Possible explanations for these unexpected findings might include that the limited number of heavy alcohol users in the sample as mentioned previously, or benefits of perinatal mother to child transmission (PMTCT) programs may be attenuating the synergistic effects of alcohol and HIV on gestational outcomes among HIV-infected mothers, nearly all who were on antiretroviral treatment. South Africa has successful PMTCT programs which have provided substantial HIV prevention benefits to both mothers and babies in South Africa. There may still be a synergistic negative effect of HIV and alcohol on the long-term developmental outcomes of children but this needs further investigation with larger samples of mothers with diverse adherence behaviours as well possible purposeful sampling of mothers using alcohol on the more moderate to severe end of the spectrum. Such type of future sampling would allow further exploration of the relationship between HIV status, varying ART adherence, and alcohol on gestational outcomes. Studies from elsewhere in Southern Africa indicate that antiretroviral treatment (ART) given to prevent mother-to-child transmission attenuates the negative effects of maternal HIV on fetal outcomes. For example, recent retrospective cohort data from a study in Malawi showed that HIV-infected children had significantly poorer health outcomes (development and functioning) than their uninfected peers [43].

### Limitations

We recognize several study limitations that offer several areas for development in future studies. First, this study uses self-report for alcohol data. Social reporting bias may be an issue especially for recent alcohol use. This may be due in part, to low literacy which necessitated field-worker administered assessments. Although fieldworkers were trained carefully in sensitive interviewing techniques, future studies could be aided by audio computer assisted self-interviewing (A-CASI) techniques which has been shown to improve accuracy of reporting on sensitive behaviors including substance use [44, 45]. Improved methods for gathering substance use may also address possible issues of under-reporting due to social desirability bias so the use of audio computer assisted self-interviewing methods or bio-verifiable methods for use may be warranted. Future studies could also be aided by the use of biomarker verification of alcohol [46]. Self-report on other drugs use are extremely low in this community. While alcohol has relative social acceptability, mothers, particularly in the context of pregnancy remain reluctant to volunteer this information. Second, longitudinal data would have enhanced our ability to assess the temporal relationship between maternal HIV and alcohol use. Findings indicated that mother’s HIV infection did not seem to adversely affect intra-uterine growth in regards to weight, length, head circumference, and gestational age. Future longitudinal studies could further develop our understanding of the role alcohol plays in risk of HIV infection among women desiring safe conception. Additional research should further explore why and how HIV infection might be linked to alcohol severity scores, and how differential alcohol use might impact ART adherence. The concerning levels of recent alcohol among HIV-infected mothers also suggests that future studies need to explore the temporal association between HIV, alcohol, and psychosocial outcomes among HIV-infected mothers, especially in relation to gestational outcomes. A particularly important focus of future studies is a better understanding of long-term growth outcomes among children born to HIV-infected mothers who are drinking at more severe levels. Even if children are not infected, or do not demonstrate poor gestational outcomes, there may be long-term developmental, growth, and psychosocial implications of maternal alcohol use among HIV-infected mothers. Despite these limitations which highlight areas to develop for future research, there are several strengths to this study. These include a population-based sample, use of dyadic data, and exploration of the effect of maternal HIV and alcohol use on gestational outcomes among a high risk population.

### Summary and conclusions

Although the broader literature suggests that HIV and alcohol have detrimental synergistic effects on the mortality and morbidity of individuals living with HIV, findings from this study indicate that HIV exposure alone in this cohort does not confer risk of poor growth outcomes at birth. The effect of maternal HIV and alcohol use on gestational may be attenuated by ARTs with similar findings from several recent meta-analyses and systematic reviews showing good maternal and infant outcomes for HIV-infected mothers on ARTs [47, 48]. While such findings are positive, they do not negate the potentially harmful effects of HIV and alcohol on long-term developmental outcomes of children, which need to be explored further. Our study highlights the importance of South Africa’s robust program of PMTCT of HIV. PMTCT is critical for supporting healthy mothers and protecting infants during the gestational period. However, mothers and their infants from high risk communities remain vulnerable to contextual factors which may impact individually and synergistically on gestational and developmental outcomes and meeting the needs of families is likely to require a broad approach.

While continued investment in PMTCT has provided immense benefits for mothers and children, and prevented adverse effects of HIV on mothers and their infants, additional investment is needed to develop interventions and provide policy and programmatic support to address the complex challenges faced by HIV-infected mothers including alcohol and nicotine use, poor mental health, and low SES. Indeed, our study highlights that these families face multiple risks that necessitate a strategic and intensive family approach that links together complementary intervention approaches to create what experts have termed, “a universal lattice of protection” [49]. At the foundation of these approaches is the understanding of HIV as a family disease that takes place against the complicated backdrop of overlapping epidemics of substance use, poor mental health, and poverty. Intervening at critical time points in the family trajectory, such as during gestation and early birth, offer important opportunities for prevention and building family resilience.

## Abbreviations

HIV: human immunodeficiency virus
ART: antiretrovirals treatment
PLWH: people living with HIV
FAS: fetal alcohol syndrome
PFAS: partial fetal alcohol syndrome
DCHS: Drakenstein Child Health Study
EPI: expanded program for immunization
SES: socioeconomic status
WHO: World Health Organization
ASSIST: the alcohol, smoking and substance involvement screening test
BDI-II: The Beck Depression Inventory-II
PMTCT: perinatal mother to child transmission
A-CASI: audio computer assisted self-interviewing
